# The evolution of the therapeutic concept ‘GIP receptor antagonism’

**DOI:** 10.3389/fendo.2025.1570603

**Published:** 2025-05-21

**Authors:** Frederikke Koefoed-Hansen, Mads Marstrand Helsted, Hüsün Sheyma Kizilkaya, Asger Bach Lund, Mette Marie Rosenkilde, Lærke Smidt Gasbjerg

**Affiliations:** ^1^ Center for Clinical Metabolic Research, Gentofte Hospital, Hellerup, Denmark; ^2^ Department of Biomedical Sciences, Faculty of Health and Medical Sciences, University of Copenhagen, Copenhagen, Denmark; ^3^ Department of Clinical Medicine, Faculty of Health and Medical Sciences, University of Copenhagen, Copenhagen, Denmark; ^4^ Clinical Research, Steno Diabetes Center Copenhagen, University of Copenhagen, Herlev, Denmark

**Keywords:** GIP -glucose-dependent insulinotropic polypeptide, antagonism, obesity, diabetes, pharmacology

## Abstract

Glucose-dependent insulinotropic polypeptide (GIP) is an intestinal hormone that potentiates glucose-induced insulin secretion in the postprandial state. GIP exerts a broad range of other physiological actions e.g. in the pancreas, bone tissue, and vasculature. In more than 20 years, GIP receptor antagonism has contributed to the discoveries of the role of GIP within both human and animal physiology. In 1986, a fragment of the biological active bovine GIP(1-42), was discovered and characterized as the first GIP receptor antagonist. Several different molecules have been identified, including peptides, vaccines against GIP, GIP antibodies, and antibodies against the GIP receptor. Today, GIP receptor antagonists are not only used as scientific tools but due to significant metabolic effects, they also have a therapeutic purpose. The beneficial clinical effects of GIP receptor antagonism are supported by comparable phenotypic traits of individuals with loss-of-function genetic receptor variants. Novel insights into GIP receptor targeting treatment reveal that both GIP receptor antagonists and agonists, when combined with glucagon like peptide 1 (GLP-1) receptor activation, are associated with improved glycemic control and weight loss. This paradoxical scenario highlights the complexity of GIP receptor pharmacology. Moreover, the long-term effects of therapeutic GIP receptor antagonism in humans are not fully elucidated and are thought to depend on the specific drug molecule, receptor functions, and the extent of GLP-1 receptor activation. With this review, we provide an overview of the preclinical and clinical evidence of GIP receptor antagonism from the central early findings to the current therapeutics in clinical development. Finally, the current therapeutic developments and the further therapeutic potential within GIP receptor antagonism are discussed.

## 1 Introduction

### 1.1 Physiological actions of GIP

Glucose-dependent insulinotropic polypeptide (GIP) is a 42 amino acid-peptide and gut-derived hormone. GIP is produced by enteroendocrine K cells (1) found in the proximal part of the small intestine but also located throughout the gastrointestinal tract to a lower extent (2). In the fasting state, the plasma levels of GIP are relatively low (3), but measurable concentrations of GIP are present throughout the entire day and night (4). Food consumption leads to a rapid rise in GIP secretion, and several nutrients have been confirmed as GIP secretagogues: glucose, amino acids, short- and long-chain fatty acids, and bile acids (5–8).

**Figure 1:**
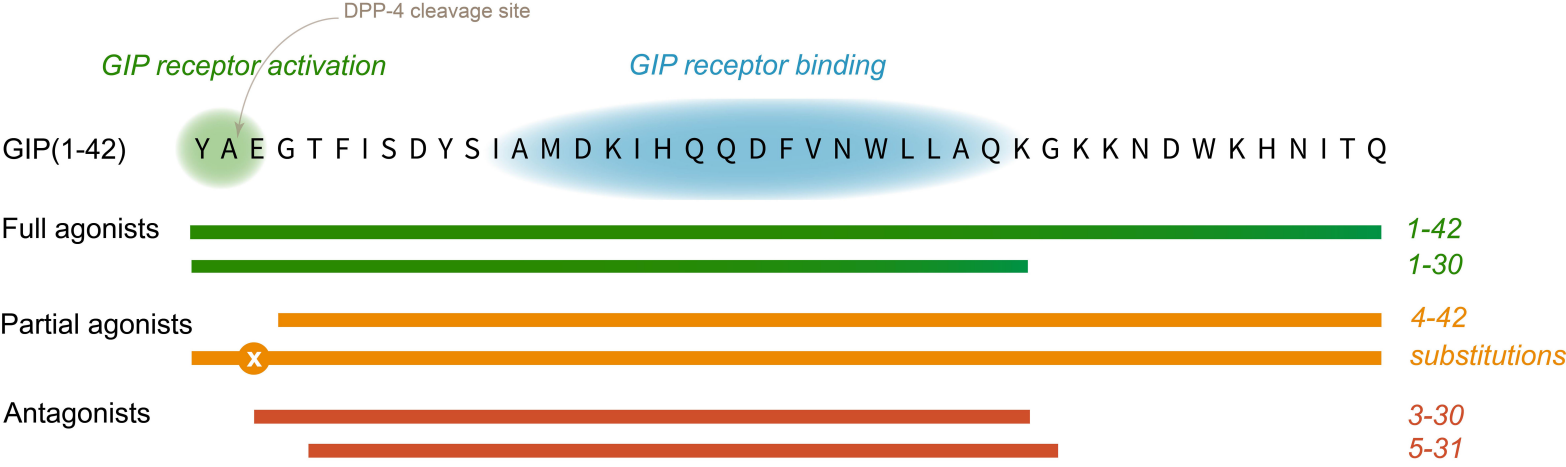
Peptide sequence and bioactive regions of GIP. The N-terminus is essential for GIP receptor activation, whereas the mid-region is crucial for binding to the receptor. Therefore, GIP receptor agonist peptides based on the natural sequence includes the N-terminus. If removed or modified by amino acid substitutions e.g. at position 3, the peptide can be a partial agonist. If the N-terminus is removed and the mid-region retained, the peptide has antagonistic properties. DPP-4, di-peptidyl-peptidase 4; GIP, glucose-dependent insulinotropic polypeptide.

The biologically active GIP(1-42) is short-lived (half-life of 7 minutes) (9). In the circulation and in the liver and kidneys, GIP(1-42) is inactivated by the enzyme dipeptidyl peptidase 4 (DPP-4) through cleavage of the two N-terminal amino acids (**Figure 1**). The degradation product is the inactive GIP(3-42), which constitutes the majority of the circulating GIP variants (10). Another variant of GIP is the C-terminally truncated form, GIP(1-30)NH_2_. GIP(1-30)NH_2_ is derived from the cleavage of the precursor protein pro-GIP by prohormone convertase 2 (11), and circulates in low picomolar concentrations (12). GIP(1-30)NH_2_ activates the GIP receptor with equivalent potency and efficacy as GIP(1-42) and elicits the same physiological effects on glucose- and bone metabolism in humans (13). Due to the identical amino acid sequence in the first 30 amino acids, GIP(1-30)NH_2_ also acts as a substrate for DPP-4 (**Figure 1**). The cleavage of GIP(1-30)NH_2_ subsequently results in GIP(3-30)NH_2_, a fragment that binds to the human GIP receptor with high affinity but does not have any intrinsic activation of the human GIP receptor in physiological concentrations (14).

The human GIP receptor can be found in multiple organs and tissues, reflecting its various physiological functions (15). GIP is an incretin hormone that, along with glucagon-like peptide 1 (GLP-1), postprandially potentiates glucose-stimulated insulin secretion from pancreatic beta cells (16). Additionally, GIP is suggested to regulate glucose homeostasis by stimulation of pancreatic alpha cells to secrete glucagon during euglycemia and hypoglycemia (17). A key physiological difference between the two incretin hormones, GIP and GLP-1, is therefore, that GIP is able to stimulate glucagon secretion, whereas GLP-1 suppresses glucagon secretion (18).

Beyond the pancreatic islet cells, the GIP receptor is expressed within the vascular system, including the heart and various endothelial cells. Here, GIP receptor activation may contribute to the increase in heart rate and reduction in arterial blood pressure observed after GIP administration (19, 20). Additionally, GIP is suggested to increase blood circulation in adipose tissue (21, 22) and the gastrointestinal tract (23, 24).

In bone, exogenous and endogenous GIP is found to decrease bone resorption by reducing osteoclast activity and improving osteoblast survival (25–27). In addition, GIP administration increases markers of bone formation (20, 28, 29). Hereby, GIP appears to uncouple the otherwise parallel process of bone remodeling, ultimately resulting in a beneficial impact of GIP on bone metabolism (20, 26).

Finally, infusion of GIP increases the deposition of triacylglycerol in adipose tissue (22). This is thought to be due to the GIP-induced increase in blood flow to subcutaneous adipose tissue and enhanced triglyceride clearance (22). However, although primarily assessed in animal models, the direct effects of GIP in adipocytes and adipose tissue fibroblasts are also possible (30–32). GIP receptor expression has been detected in both human and mouse adipocytes (33, 34), and GIP receptor activation in adipose tissue could protect mice from obesity and induce weight loss (32).

### 1.2 GIP receptor signaling pathways

**Figure 2:**
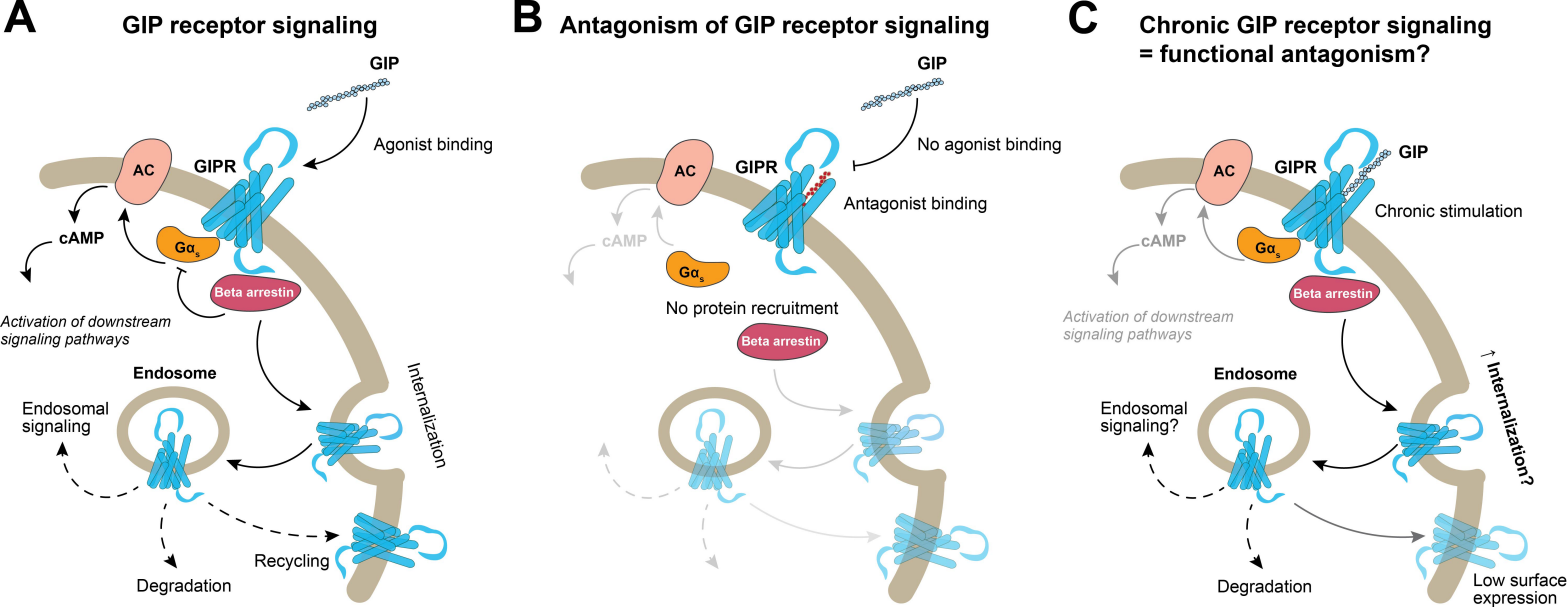
GIP receptor signaling. **(A)** The GIP receptor is activated upon binding of GIP, which leads to the coupling of Gα_s_, and subsequent activation of adenylyl cyclase, that in return increases intracellular cAMP concentrations and initiate further downstream signaling pathways. The recruitment of beta arrestin inhibits Gα_s_ coupling and promotes receptor internalization, leading to intracellular signaling from endosomes, receptor recycling or degradation; **(B)** A GIP receptor antagonist will block these pathways; **(C)** Long-term/chronic GIP receptor signaling could enhance receptor internalization and desensitization, leading to a reduced number of receptors available on the cell surface thereby introducing *functional antagonism* as a response to GIP agonist administration. GIP, glucose-dependent insulinotropic polypeptide; AC, adenylyl cyclase; cAMP, cyclic adenosine monophosphate.

The actions of GIP are mediated by the GIP receptor, a G protein-coupled receptor (GPCR) in the class B1 receptor family (35). Activation of the GIP receptor involves ligand binding, leading to conformational changes in the receptor that subsequently initiates intracellular signaling cascades (36). Upon ligand binding that leads to GIP receptor activation, the receptor primarily couples to the Gα_s_ subfamily. When the G protein is coupled to the GIP receptor adenylyl cyclase is activated to increase the intracellular concentrations of the secondary messenger, cyclic adenosine monophosphate (cAMP) (**Figure 2A**). An increased concentration of cAMP leads to the activation of protein kinase A (PKA), which activates tissue-specific downstream signaling pathways (37). Adding more complexity to the interpretation of GIP receptor pharmacology, the conformational shift in the receptor also facilitates the recruitment of beta arrestin. The beta arrestin recruitment is necessary for GIP receptor function (30, 38) as it desensitizes the receptor by hindering binding to its corresponding G protein and hereby hinders the intracellular signaling cascade. Moreover, beta arrestin recruitment is essential for GIP receptor internalization where the GIP receptors are removed from the cell surface (30). Subsequently, the receptor is taken up by endosomes, where it can either continue its signaling intracellularly, be recycled to the cell surface (resensitization) or undergo lysosomal degradation. This indicates that receptor signaling is tightly regulated by beta arrestins (39). In addition, with sustained stimulation, there will be fewer receptors available on the cell surface due to receptor desensitization and internalization. Hence, with fewer receptors available for targeting chronic stimulation with agonists, is proposed to act as *functional antagonism* of the GIP receptor (40–42) (**Figure 2C**).

### 1.3 GIP receptor antagonism

**Figure 3:**
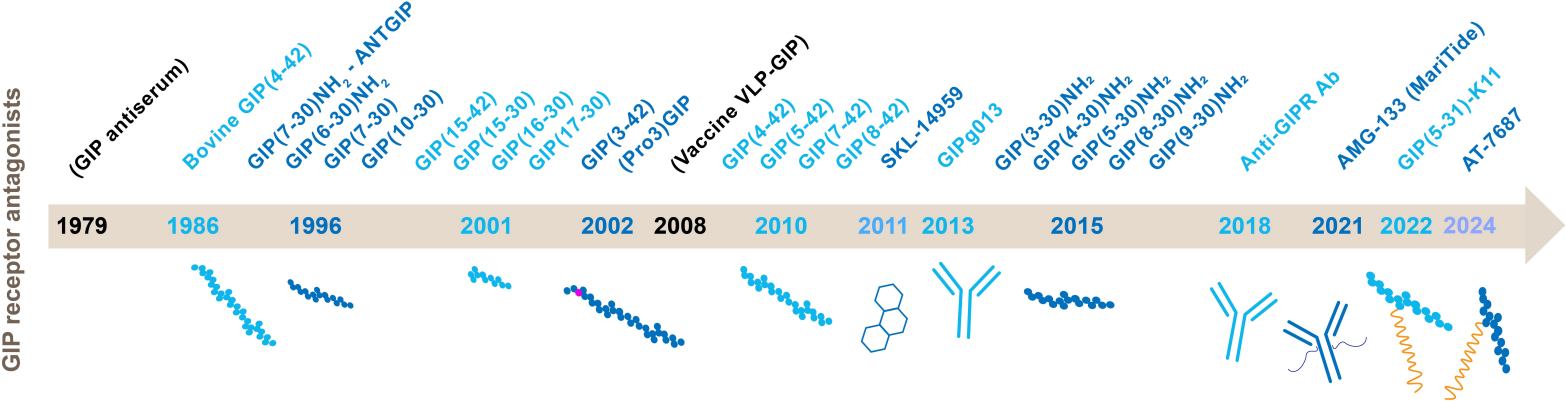
Timeline of major discoveries of GIP receptor antagonists. GIP, glucose-dependent insulinotropic polypeptide; VLP, virus-like peptide.

The physiology of GIP has been explored extensively by exogenous GIP infusions resulting in both physiological and supraphysiological concentrations (43–47). However, these findings may not fully reflect the role of endogenous GIP. To understand the physiological effects of endogenous GIP receptor activation, the development of GIP receptor antagonists has been essential. The first GIP receptor antagonist was introduced in 1986 (**Figure 3**), and in recent years, the truncated and deactivated form of GIP, GIP(3-30)NH_2_, has proven to be a potent and specific GIP receptor antagonist in humans in high concentrations, and suitable for studies of endogenous GIP physiology and pathophysiology (14) (**Table 1**). In parallel with the human studies, preclinical and *in vitro* pharmacological work has played a crucial role in understanding of the physiological and pathophysiological role of endogenous GIP and the potential of GIP receptor antagonism as a therapeutic concept. Various methods to inhibit the GIP system in rodents result in reduced body weight and improve insulin sensitivity (52). Combined with the theory of GIP being an obesogenic hormone (53), these prior findings have highlighted the potential of GIP receptor antagonism, and today the development of GIP receptor antagonists has also emerged as an attractive therapeutic approach for addressing obesity and cardiovascular diseases (54, 55). In the following, we review the preclinical and clinical evidence of GIP receptor antagonism.

**Table 1:** *In vitro* results for the most potent and widely used GIP receptor antagonists.

GIP receptor antagonists	Inhibition of GIP(1-42)-induced receptor activation Potency/IC_50_ (nmol/l)	GIP receptor binding Affinity/K_d_ or K_i_ (nmol/l)
GIP(3-42) (14, 48)	138	22
SKL-14959 (49)	2,900	55
GIP(3-30)NH_2_ (14)	2.3	3.4
GIP(5-30)NH_2_ (14)	5.9	5.9
AMG133 (50)	136	Not Available
AT-7687 (51)	9.5	2.0

The results were not performed in parallel and originate from various *in vitro* assays. GIP, glucose-dependent insulinotropic polypeptide; IC_50_, half-maximal inhibitory concentration; K_d,_ dissociation constant; K_i_, inhibition constant.

## 2 Development of GIP receptor antagonists

GIP was identified in the 1970s (56) and first described as an enterogastrone that inhibited gastric acid secretion in dogs, and, thus, initially named *gastric inhibitory peptide* (57). A few years later, GIP was characterized as an insulinotropic gut-derived peptide hormone, and the name was changed to *glucose-dependent insulinotropic polypeptide* (56). In 1979, intravenous infusions of a GIP antiserum in rats were able to inhibit the action of GIP on glucose-induced insulin release, hereby, showing the first inhibition of GIP activity (58) (**Figure 3**).

In 1986, the biological activity of bovine GIP(1-42) and several of its fragments was characterized according to their ability to promote insulin secretion from an isolated perfused rat pancreas. The fragment bovine GIP(4-42) was a partial agonist of the rat GIP receptor, concluding that N-terminal truncation of GIP alters its pharmacological properties from full agonist to partial agonist (59). This called for further studies investigating modification of the GIP C-terminus on interaction with the GIP receptor.

Ten years later, in 1996, various peptide fragments of GIP were studied to investigate the potential for identifying a GIP receptor-specific antagonist. GIP(7-30)NH_2_ was found to be a GIP-specific receptor antagonist that inhibits GIP-stimulated cAMP production and insulin release in a concentration-dependent manner. Due to the compelling findings of antagonistic properties, GIP(7-30)NH_2_ was named ANTGIP (60). In 1999, infusions of GIP(7-30)NH_2_ in rats, reduced insulin release after an intragastric glucose meal. This demonstrated the antagonistic effect while also providing evidence for the crucial role of GIP receptor signaling in potentiating the insulin response to oral glucose (61).

Alongside studies of GIP(7-30)NH_2_, other truncated forms: GIP(10-30), GIP(6-30)NH_2_ and GIP(7-30) were characterised as GIP receptor antagonists with the ability to block GIP’s activation of the GIP receptor *in vitro*. Of the three, GIP(6-30)NH_2_ was the most potent antagonist followed by GIP(7-30) and finally GIP(10-30), ranked by their potencies as inhibitors of GIP-stimulated cAMP production (62).

In 2001, several synthetic peptide GIP fragments were tested to identify the molecule’s bioactive domain. Here, the GIP fragments GIP(15-42), GIP(15-30), GIP(16-30) and GIP(17-30) were characterized as weak antagonists at the human GIP receptor *in vitro*, and the amino acids number 6-30 are required for effective GIP receptor antagonism as a high-affinity binding domain (63) (**Figure 1**). The following year, the important role of the N-terminus in GIP’s full biological activity was again demonstrated. *In vitro*, GIP(17-30) and GIP(4-42) did not increase cellular cAMP production and had only weak insulin releasing activity compared with the native GIP (64).

Simultaneously, the most abundant circulating GIP fragment GIP(3-42) was identified as a GIP receptor antagonist with the ability to moderate GIP-induced cAMP production and insulin secretion *in vitro* (65) (**Table 1**). However, in 2006 it became clear that GIP(3-42) does not function as a physiological antagonist *in vivo* as its maximal circulating levels are insufficient to elicit antagonistic effects (48).

At the same time, in 2002, *Gipr* knockout mice were found to be protected against both obesity and insulin resistance induced by high-fat feeding (53). These findings led to a suggested role for GIP in the development of obesity, highlighting the therapeutic opportunity of GIP receptor antagonism, and subsequently leading to great interest and research activity within the field (53).

Also in 2002, a GIP analogue with an N-terminal substitution of glutamic acid in position 3 with proline (Pro3), was made as an attempt to prolong the GIP actions by protecting from DPP-4 degradation (66) (**Figure 1**). In animal models, (Pro3)GIP was found to be a potent enzyme-resistant GIP receptor antagonist by its ability to block GIP’s effects *in vitro* and *in vivo* (66, 67). However, years later in 2016, a pharmacological analysis of this ligand demonstrated interspecies differences between the rodent and human GIP system and also led to the conclusion that the human Pro3(GIP) was a potent partial agonist with the ability to activate the human GIP receptor with a maximal efficiency (E_max_) of 90% compared to human GIP (68). This finding annulled its clinical potential as a GIP receptor antagonist.

In 2008, an immunization approach, led to the development of an active vaccine with GIP peptides covalently attached to virus-like particles (VLP-GIP). Vaccination of mice with VLP-GIP produced high titers of specific antibodies, that bound to GIP and prevented activation of the GIP receptor. As seen for the GIP receptor knockout mice, VLP-GIP efficiently reduced body weight gain in animals on a high-fat diet (69), supporting that the GIP receptor plays a role in developing obesity.

Two years later in 2010, GIP(4-42), GIP(5-42), GIP(7-42) and GIP(8-42) also proved to have functionally interesting antagonistic effects at the GIP receptor *in vitro* (70). However, only GIP(8-42) exhibited antagonistic properties *in vivo* in mice reducing the effects of the native agonist, GIP(1-42).

In 2011, a small molecule, low-molecular GIP receptor antagonist, SKL-14959 (49), was studied both *in vitro*, and *in vivo* by injections in mice. The small molecule has a strong affinity for the GIP receptor and inhibition of GIP-stimulated cAMP production *in vitro* (**Table 1**). Furthermore, SKL-14959 suppresses insulin secretion and reduces lipolytic effects *in vivo* (71).

In 2013, Gipg013, a GIP receptor immunoglobin G antibody, was presented. Gipg013 is a specific competitive antagonist with equally high potency across mouse, rat, dog and human GIP receptors (72). *In vivo*, Gipg013 dose-dependently reduces GIP-induced insulin secretion and was one of the first GIP receptor antagonizing antibodies published (72). The following year, antagonism of the GIP receptor by palmitoylation of GIP analogues with N- and C-terminal modifications reduced body weight and improved metabolic control in high-fat-fed mice. Consequently, GIP receptor antagonism was proposed as a potential strategy for treating obesity-related diabetes (73).

Until 2015, a common limitation of the identified GIP receptor antagonists was the absence of human data, raising concerns about their potential clinical application. In 2015, N-terminally truncated variants of the C-terminally truncated and fully bioactive GIP(1-30)NH_2_ were studied. Here, GIP(3-30)NH_2_ and GIP(5-30)NH_2_ were characterized as potent, competitive and high-affinity antagonists of the human GIP receptor (14) (**Table 1**). In 2017, GIP(3-30)NH_2_ was confirmed to be highly selective for the human GIP receptor without any agonistic properties. To complement human studies, mouse GIP(3-30)NH_2_ was also confirmed as a GIP receptor antagonist with the ability to impair the glucose-lowering effects of human GIP(1-42) in mice (74). In 2017, the first human study with GIP(3-30)NH_2_ was performed. GIP(3-30)NH_2_ demonstrated an inhibition of the GIP-potentiated glucose-stimulated insulin secretion by 82%. Importantly, the infusions were well tolerated and did not trigger any adverse effects (30). That same year, human infusions of GIP(3-30)NH_2_ were also used to block the previously observed effects of exogenous GIP on adipose tissue perfusion (22). In conclusion, GIP(3-30)NH_2_ was defined as a new scientific tool to investigate GIP physiology (75). Over the following years, in several human studies, GIP(3-30)NH_2_ was administered intravenously to study the effects of endogenous GIP by inhibiting the GIP receptor and comparing the results with placebo infusions (76). GIP(3-30)NH_2_ has been infused in both healthy individuals (16, 22, 24, 75), patients with type 1 diabetes (77), type 2 diabetes (78, p. 2) with or without obesity (79), acromegaly (80), before and after gastric bypass surgeries (79), and in totally pancreatectomized patients (81). Species variants of GIP(3-30)NH_2_ (for especially mice and rats) are also selective and efficient tools (30, 82, 83), and with 15 completed human studies and several ongoing, GIP(3-30)NH_2_ has become the most widely used tool for studying the role of the GIP system in humans.

In 2018, another non-peptide candidate for the inhibition of the GIP receptor was presented using an antibody-based approach. In mice, treatment with anti-murine GIP receptor antibody (muGIPR-Ab) protected against body weight gain (54). This finding was replicated in nonhuman primates using another antibody, now against the human GIP receptor, supportive of an antagonistic approach for obesity treatment (54).

In 2021, AMG133, a bispecific molecule that combines GIP receptor antagonism and GLP-1 receptor agonism was presented. The molecule is a human monoclonal anti-human GIP receptor antibody conjugated with two GLP-1 receptor agonist peptides. In preclinical efficacy studies in diet-induced obese (DIO) mice and obese monkeys, treatment with AMG133 reduces body weight and improves metabolic parameters to a larger extent than a GLP-1 receptor agonist alone (50). Additionally, AMG133 has an extended pharmacokinetic profile with a half-life of up to 9.1 days in obese monkeys, suitable for less frequent dosing regimens (50). Phase 1 and phase 2 studies of the clinical trial development program for AMG133 are completed with results from phase 1 publicly available (84), while, at this time, phase 2 data have only been disclosed through a company announcement with headline results (85). In 2022, a GIP(5-31) analogue was discovered and proved to function as a potent GIP receptor antagonist (86). *In vitro*, a GIP(5-31) analogue palmitoylated at a lysin in position, GIP(5-31)-K11, inhibited GIP-induced cAMP production in both human and rodent systems. *In vivo*, the GIP(5-31) analogue inhibited GIP-stimulated insulin secretion in human islets. Additionally, co-administration of this antagonist with a GLP-1 receptor agonist in DIO mice potentiated weight loss compared to either monotherapy (86).

Latest in 2024, AT-7687, an optimized peptide analogue of the naturally occurring GIP(3-30)NH_2_ was characterized as a potent and selective GIP receptor antagonist (**Table 1**) with a circulatory half-life of 27.4 hours (51) (**Figure 3**). The *in vivo* effects of AT-7687 were evaluated in obese cynomolgus monkeys, and when combined with the GLP-1 analogue liraglutide, AT-7687 enhances weight reduction induced by liraglutide and improves glycemic control and lipid metabolism (51).

## 3 Results of reduced GIP receptor activation in humans

**Figure 4:**
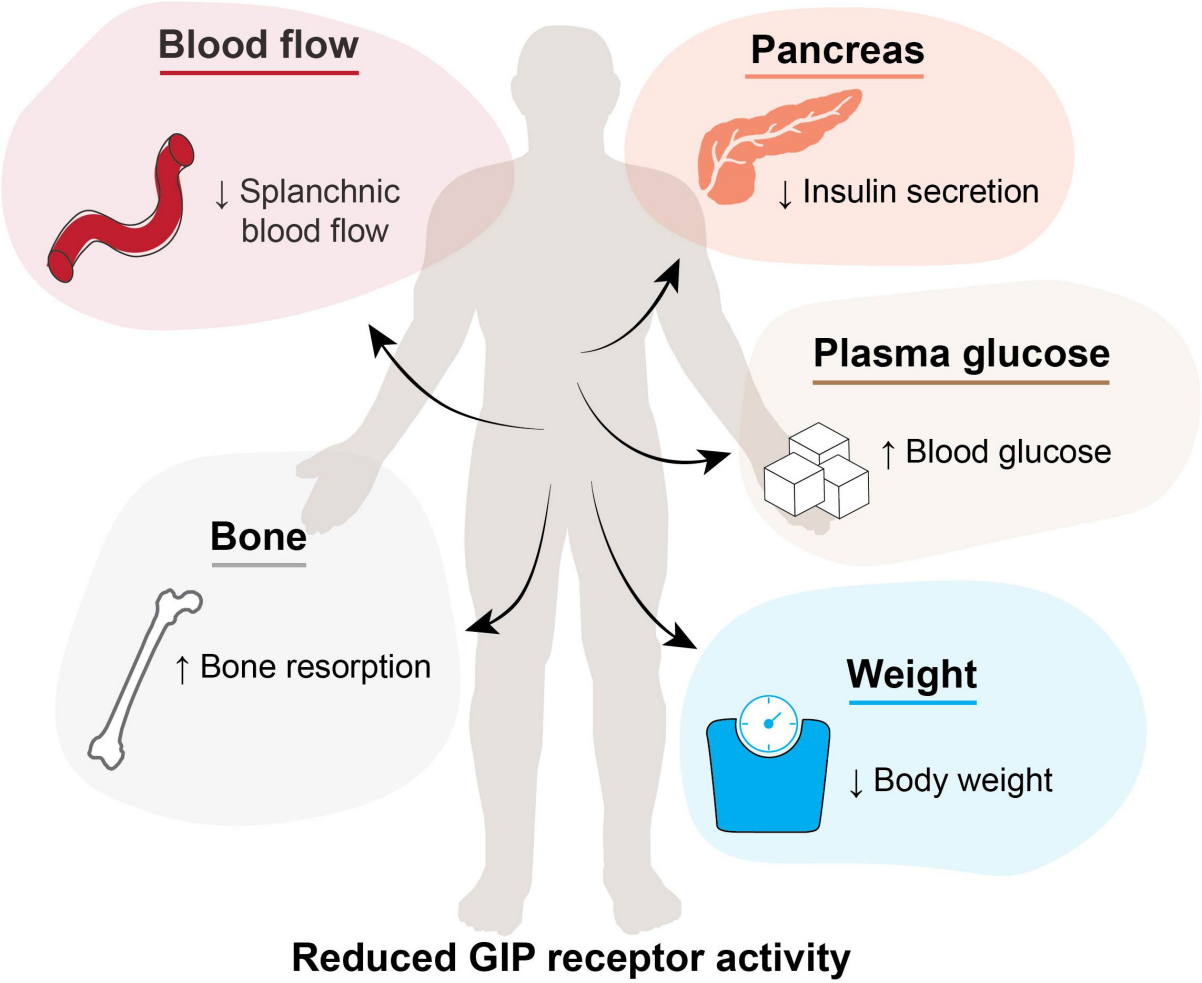
Effects of GIP receptor inhibition or genetic loss-of function receptor variants in humans. Reduced GIP receptor activity leading to reduced splanchnic blood flow, reduced insulin secretion, increased blood glucose, increased bone resorption and reduced body weight. GIP, glucose-dependent insulinotropic polypeptide.

Some GIP receptor antagonists have contributed to the elucidation of the physiological properties of endogenous GIP. Moreover, several GIP receptor antagonists have also been used to evaluate the therapeutic potential of inhibiting endogenous GIP actions. Among the described antagonists, GIP(3-30)NH_2_ has been particularly instrumental in advancing the understanding of the human physiology of the GIP system, whereas modified peptides and antibodies have contributed with therapeutic perspectives. Another relevant contribution to the elucidation of GIP biology is studies of genetic loss-of-function variants of the human GIP receptor. These studies have provided valuable insights into the potential long-term effects of GIP receptor antagonism, and offered a clearer understanding of how impairing GIP receptor activity may impact physiological traits related to GIP’s biological effects. In the following, we describe and discuss the effects of reduced or abolished GIP receptor activation within different organs and tissues in humans (**Figure 4**).

### 3.1 Glucose metabolism

GIP(3-30)NH_2_ administration has yielded significant outcomes within glucose metabolism across human studies. In healthy individuals, GIP(3-30)NH_2_ infusions inhibit GIP-potentiated glucose-stimulated insulin secretion (75), thereby confirming GIP’s role as a potent incretin hormone (16, 87). In addition, infusions of GIP(3-30)NH_2_ induce higher postprandial plasma glucose levels (16, 87).

In individuals with type 2 diabetes, GIP(3-30)NH_2_ infusions decrease insulin secretion, with little effect on postprandial glycemia (78). These findings reveal an insulinotropic effect of endogenous GIP in individuals with type 2 diabetes. However, this contrasts to findings from earlier infusions of exogenous GIP(1-42), which failed to enhance insulin secretion in individuals with type 2 diabetes (88, 89). The lack of insulinotropic response to GIP has long been claimed as the reason for not targeting the GIP receptor in this patient group. Although later, endogenous GIP was revealed to have significant insulinotropic properties. Furthermore, several novel therapeutic compounds (GIP receptor antagonists and agonists including multiple-targeting compounds) have also shown beneficial glucometabolic and body weight lowering effects in individuals with type 2 diabetes and obesity (40, 41). Highlighting the complexity of the GIP system in humans, conclusions based on short-term GIP(1-42) infusions failed to demonstrate its therapeutic potential.

We recently assessed the role of endogenous GIP in individuals with obesity during fasting using GIP(3-30)NH_2_. Here, we found infusions of GIP(3-30)NH_2_ to cause higher plasma glucose levels, indicating a glucose-lowering effect of endogenous GIP. However, GIP(3-30)NH_2_ did not affect insulin secretion in the fasting state (90). A possible explanation for these findings is that endogenous GIP may play a role in the regulation of insulin sensitivity in humans, similar to observations in mice (91).

In gastric bypass-operated individuals with obesity and no history of diabetes, GIP(3-30)NH_2_ does not affect insulin secretion nor plasma glucose levels (79). These results differ from the effects of GIP(3-30)NH_2_ in non-operated individuals, and points to a less important role of GIP in glucose metabolism after gastric bypass surgery (79, 92). However, the effect of GIP(3-30)NH_2_ still appears to differ depending on the type of gastric surgery. Following gastric sleeve, infusions of GIP(3-30)NH_2_ induce a slight increase in plasma glucose levels, whereas no changes are found after Roux-en-Y gastric bypass. Therefore, endogenous GIP seems to retain a role in postprandial glucose regulation following gastric sleeve but when the first part of the small intestine is bypassed (Roux-en-Y gastric bypass) endogenous GIP does not contribute to the postprandial glucose regulation (79). In totally pancreatectomized individuals, GIP(3-30)NH_2_ infusions do not affect postprandial plasma glucose levels highlighting the importance of the pancreas in GIP’s glucose-regulating effects (81).

Infusions with GIP(3-30)NH_2_ in healthy individuals during a mixed meal test or an oral glucose tolerance test as well as in persons with obesity in the fasting state do not affect glucagon levels (16, 87, 90). However, in individuals with type 2 diabetes, infusions of GIP(3-30)NH_2_ reduced glucagon concentrations (78), illustrating the potential of GIP receptor antagonism to limit the paradoxical postprandial hyperglucagonemia in individuals with type 2 diabetes (93).

Treatment with AMG133 in individuals with obesity decreases HbA1c levels and reduces fasting glucose levels in a dose-responsive manner (84). In DIO mice and obese monkeys, AMG133 decreases fasting insulin levels, but this effect was not seen in the clinical trial of AMG133 (84). Furthermore, AMG133, in higher doses, induces lower fasting glucagon levels compared to placebo (84). The results of the phase 1 studies of AMG133 could provide insights into the role of GIP receptor antagonism within glucose metabolism, however, determining the exact effect of the GIP receptor antagonism remains challenging due to an anticipated influence of GLP-1 receptor agonism.

### 3.2 Adipose tissue

In humans, infusions of GIP(3-30)NH_2_ during a hyperinsulinemic and hyperglycemic clamp reduce GIP-induced blood flow to subcutaneous adipose tissue and inhibit the increase in triacylglycerol clearance observed with GIP administration (22). Furthermore, the administration of GIP(3-30)NH_2_ increased free fatty acid (FFA) output and a higher FFA/glycerol-ratio, suggesting a reduction in *in-situ* re-esterification of FFA and lipolytic activity (22). However, these findings are based on the inhibition of administered GIP, and, therefore, they do not describe the clinical effect of GIP receptor antagonism and inhibition of endogenous GIP in adipose tissue.

Nevertheless, GIP receptor antagonism is thought to exert beneficial metabolic effects due to the expression of GIP receptors on adipocytes (94), along with elevated circulating GIP levels observed in both individuals with obesity (95) and individuals given a high-fat-diet (96). Therefore, reducing fat deposition through GIP receptor antagonism still hold promising therapeutic potential supported by animal models and the fact that individuals with loss-of-function *GIPR* genetic variants have a lower body mass index (BMI) (39, 97–99).

### 3.3 Bone metabolism

Addressing the acute changes in the postprandial state, GIP(3-30)NH_2_ infusions diminished GIP-induced suppression of the bone resorption marker carboxy-terminal type 1 collagen crosslinks (CTX) in both healthy individuals (26) and individuals with type 2 diabetes (78). Furthermore, GIP(3-30)NH_2_ has been used to demonstrate an uncoupling of the otherwise coupled processes of bone resorption and formation assessed by a decrease in CTX and a temporary increase in the bone formation marker procollagen type 1 amino terminal propertied (P1NP) in healthy individuals (100). Characterizations of individuals with genetically impaired GIP receptor function corresponds with the findings following GIP receptor agonist or antagonist administrations. Three amino acid-altering GIP receptor variants (R190Q, E288G, and E354Q) are all associated with lower bone mineral density, and in postmenopausal women followed for 10 years, who were homozygous carriers of the E354Q variant, showed a higher risk of non-vertebral fractures (99, 101). However, a recent large meta-analysis showed no association between these three variants and low bone mineral density or fracture prevalence (102).

### 3.4 Appetite regulation and weight management

Similar to animal studies with deletion of the *GIPR* gene that exhibit protection toward obesity (53), early genome-wide association studies (GWAS) in humans have revealed the *GIPR* gene to be associated with BMI (103). Specifically, carriers of *GIPR* variants that are pharmacologically classified as loss-of-function variants with reduced cell surface expression, cAMP production, beta arrestin 2 recruitment, internalization, and endosomal signaling have consistently been associated with lower adiposity-related traits, including BMI (103) and nominally lower prevalence of obesity (39, 97–99). However, studies with the available GIP receptor antagonists have not shown an acute effect on appetite regulation in humans (16) assessed by an *ad libitum* meal test and visual analogue scale questionaries (16, 87). When tested in obese cynomolgus monkeys, treatment with AT-7687 in combination with liraglutide results in lower total energy intake and significant body weight loss compared to placebo but no differences when compared to liraglutide alone. Thus, treatment with AT-7687 and liraglutide reduced appetite but, in this first preclinical study, the effect of the GIP receptor antagonist could not be separated from the GLP-1 receptor agonist alone (51). Recently, in a phase 1 clinical study, AMG133 demonstrated a pronounced dose-dependent weight loss in participants with obesity. Participants receiving multiple ascending doses of AMG133 experienced sustained weight loss for up to 150 days after the last dose (84). It is worth noting that GLP-1-induced appetite suppression is a well-established mechanism in the treatment of obesity. However, no clear mechanism behind the potential of weight loss induced by GIP receptor antagonism has been identified. While co-receptor targeting, as seen with AMG133, has shown promising results in weight reduction, it remains unclear whether this is an additive or synergistic effect, or if the main mode of action behind appetite suppression is based on GLP-1 receptor activation (104).

### 3.5 Nausea

Recent technological advancements have detected the GIP receptor in the brain of mice, specifically in inhibitory gamma-aminobutyric acid (GABA) neurons located within the area postrema, which function as a chemoreceptor trigger zone for vomiting (105). GIP receptor agonists have shown antiemetic effects and reduces nausea-like behavior in animal models (105). However, in the human brain, the presence of the GIP receptor is still elusive. Recently, administration of a long-acting GIP receptor agonist in humans, reduced GLP-1 analogue related gastrointestinal side effects, including nausea and vomiting (106). Although no findings have yet been made with GIP receptor antagonists in this field, the findings suggests that the GIP system is involved in the regulation of central nervous system-mediated anorexigenic and emetic behaviors (106).

### 3.6 Memory and other cognitive functions

As GIP receptor expression have been demonstrated in different brain regions of animals (105, 107), GIP could play a direct role in modulation aspects of brain functions. Although, there are no human data describing the effect of GIP activity on memory and other cognitive functions, several animal studies have shown that activation of the GIP receptor has a profound effect on neuronal plasticity (108). As an example, in mice, prolonged GIP receptor activation improves cognitive function by an increased recognition index (109). Furthermore, it improves hippocampal synaptic plasticity (109, 110). While the effects of GIP receptor antagonism on these outcomes remain unknow, GIPR knockout mice have been shown to exhibit impaired learning and memory, as well as reduced synaptic plasticity (111).

### 3.7 Cardiovascular effects

In a GWAS, the genetic GIP receptor variant, E354Q, and the risk of cardiovascular diseases are nominally associated (112), and also, carriers of E354Q have been associated with lower risk of coronary artery disease (113). The carriers of the loss-of-function GIP receptor variants, R190Q and E288G, display higher blood pressure and an increased tendency toward hypertension (99) but a comprehensive study of larger number of loss-of-function GIP receptor variants did not reveal similar effects on cardiovascular traits (39). The direct mechanism of the potential effects of long-term reduced GIP receptor activity in the cardiovascular system is not yet confirmed (55) but recently, infusions of GIP(3-30)NH_2_, have highlighted the crucial role of endogenous GIP in the vascular system during meal digestion. During oral glucose ingestion, GIP(3-30)NH_2_ infusion reduces postprandial splanchnic blood flow in healthy men (24). The reduced blood flow in the major gastrointestinal vessels, the superior mesenteric artery and portal vein, demonstrate that endogenous GIP contributes to postprandial hyperemia (24). However, since no measurements of splanchnic hemodynamic changes after more long-term exposure to GIP receptor antagonism have been conducted, in either animal models or humans, the therapeutic implications of this effect are uncertain.

### 3.8 Adverse effects of GIP receptor antagonism

So far, no adverse effects have been reported with the administration of GIP receptor antagonists in humans or animals. Given the established physiological effects of GIP receptor activation, GIP receptor antagonism could potentially lead to undesirable increases in blood glucose levels due to reduced insulin secretion. Additionally, alterations in lipid distribution could cause concerns about where lipids would be stored and how it would be metabolized (114). Longer treatment with GIP receptor antagonists could also reduce bone mineral density (100), and hereby increase the risk of fractures. Moreover, the reduced splanchnic blood flow could affect nutrient uptake or affect digestion (23). Finally, the expected involvement of GIP in the central nervous system (107) leads to uncertainty regarding effects on memory and cognitive functions.

When other receptor antagonists are administered, a compensatory increase in the circulating hormone levels is seen (115). However, GIP receptor antagonism has not, so far, resulted in higher or lower circulating levels of GIP (46, 75, 87). Importantly, to evaluate adverse or compensatory effects, a GIP receptor antagonist must be administered longer than those presented so far. Moreover, current studies with long-term exposure to GIP receptor antagonism have only been conducted during co-stimulation of the GLP-1 receptor which induce a drug class-specific adverse effect profile, including nausea and vomiting (84, 116). Therefore, further research is needed to determine whether prolonged GIP receptor antagonism can lead to any adverse effects.

When administered in humans, GIP receptor antagonists conjugated to GLP-1 analogues have led to mild gastrointestinal-related adverse events, including nausea and vomiting (50). Considering this, the reported side effects may be GLP-1 receptor activation-related, but the exact mechanism is still unknown.

## 4 Combinations of GIP receptor targeting treatments and GLP-1 receptor agonism

**Figure 5:**
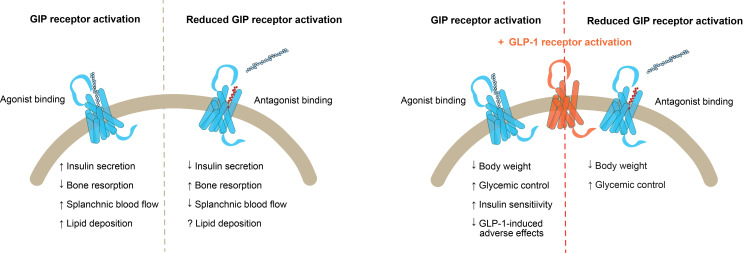
Combinations of GIP receptor targeting treatments and GLP-1 receptor agonism. Left) Outcomes of GIP receptor activation by agonist binding versus reduced GIP receptor activation by antagonist binding; Right) Outcomes of GLP-1 receptor activation in combination with GIP receptor agonist binding versus GIP receptor antagonist binding. GIP, glucose-dependent insulinotropic polypeptide; GLP-1, glucagon like peptide 1.

In recent years, GIP receptor agonists and antagonists have gained attention for their therapeutic potential in treating obesity and type 2 diabetes. Remarkably, combining GIP receptor agonism with GLP-1 receptor agonism, or GIP receptor antagonism with GLP-1 receptor agonism has led to substantial weight loss, improved glycemic control, and better cardiovascular risk profiles in both animal models and clinical trials (50, 51, 54, 84, 86, 117, 118) (**Figure 5**). However, the mechanism of action of GIP in this paradox and the extent of its role in these outcomes remains unclear. Therefore, growing interest and evidence support both stimulation and inhibition of GIP receptor signaling.

Understanding the balance between GIP receptor signaling, intracellular recycling, and desensitization is crucial for interpreting the physiological consequences of a given compound. Therefore, the traditional pharmacological terms like *agonist* and *antagonist* could be insufficient for characterizing compounds that target the GIP receptor. Today, the majority of the emerging GIP receptor targeting compounds are claimed to be agonists, and to increase insulin secretion (17, 20), to improve insulin sensitivity (91), and also, suppress appetite (119). Most of the GIP receptor targeting compounds are evaluated *in vitro* as stimulators of a single cellular pathway, which is the Gαs-mediated cAMP production (14, 30, 37). However, assessing only cAMP production may be insufficient, as e.g. beta arrestin recruitment to the GIP receptor is a key regulator of receptor signaling, particularly in post-activation trafficking, including receptor internalization, continued signaling from intracellular compartments like endosomes and recycling of the receptor back to the cell surface. Since the different ‘agonists’/compounds, all of which stimulate Gαs recruitment to the GIP receptor, have distinct pharmacology, they may affect interaction of the various effector molecules with the GIP receptor differently (39, 42) (**Figure 2**). Hence, a more thorough pharmacological characterization is necessary for each developed compound.

The GIP receptor-GLP-1 receptor dual agonist, tirzepatide, ranks among the most effective therapeutic agents within treatment of obesity and diabetes to date (118). It has demonstrated the ability to reduce body weight by more than 20% in individuals with obesity (118), and to improve glycemic control to a degree that 87-91% of enrolled individuals with type 2 diabetes reach HbA1c levels below the type 2 diabetes diagnostic threshold after 40 weeks of treatment (120). Interestingly, while tirzepatide acts as an agonist of the GLP-1 receptor, it may effectively act as a GIP receptor antagonist. Its binding to the GIP receptor induces receptor internalization which, with continuous treatment, could lead to receptor downregulation and reduced GIP signaling (121). However, clinical evidence of GIP receptor engagement is still lacking and due to similar actions of GIP and GLP-1 receptor activation, the distinct mode of action for this dual-acting compound is complex to address (41).

The only GIP receptor antagonist administered in humans for a longer period, is the aforementioned AMG133, which is an antagonistic GIP receptor antibody conjugated to two agonistic GLP-1 receptor peptide analogues (84). The mode of action for AMG133 is believed to involve this bispecific molecule simultaneously binding to both the GIP- and GLP-1 receptor, where it is demonstrated to increase cAMP production and induce internalization of co-localized GIP and GLP-1 receptors. Notably, higher levels of endosomal markers were observed in cells expressing GLP-1 receptor stimulated with AMG133, suggesting an increased localization of the receptor complex within the endosomes. This enhanced receptor internalization is, therefore, thought to amplify the effects of GLP-1 receptor agonism (50).

Besides the complex pharmacological interplay between the GIP receptor targeting ligands and the receptor addressed above, another aspect of GIP biology is the tissue-specific actions, which might affect the clinical results of GIP targeting treatments. When therapeutically targeting the GIP system, effects on other tissues than the pancreas could partly explain the paradox observed for the weight-reducing effects of GIP receptor agonism and antagonism. GIP’s action to augment splanchnic blood flow, potentially affecting nutrient uptake and its involvement in adipose tissue metabolism and suggested central affects in the regulation of appetite as seen in animal models, all remain to be further explored in humans.

## 5 Current clinical trials

In a recent press release, the results from the phase 2 study of AMG133 (*maridebart cafraglutide* or *MariTide*) are revealed to induce a substantial weight loss following 52 weeks treatment. The phase 2 study showed that participants with overweight or obesity, without type 2 diabetes had an average weight loss of up to 20% and without the occurrence of a weight loss plateau. Participants with type 2 diabetes with obesity or overweight had an average weight loss of up to 17% also without reaching a weight loss plateau. Moreover, they obtained a decrease in average HbA1c of up to 2.2 percentage points. In addition, cardiometabolic parameters were improved, including blood pressure, triglyceride levels, and high-sensitivity C-reactive protein. AMG133 was administered in longer treatment intervals with either monthly or less frequent dosing. The most common adverse effects were mild and transient gastrointestinal symptoms including nausea, vomiting and constipation. Notably, the adverse effects were mainly associated with the first dose. Due to these results, Amgen expects to initiate a phase 3 development program (85).

## 6 Conclusions

The discoveries of GIP receptor antagonists as scientific tools have elucidated the role of GIP in human physiology. The GIP receptor antagonist GIP(3-30)NH_2_ has especially contributed to a greater understanding of the GIP system in both human and rodent studies. Importantly, genetic variations within GIP receptors across different species, make the GIP system complex compared to the more conserved GLP-1 system. Thus, translation of GIP physiology and pharmacology across species can be challenging (122). Within the past years, development of GIP receptor antagonists has been an attractive therapeutic approach for combating obesity and treating type 2 diabetes. Today, more compounds that target the GIP receptor are emerging as a valuable complement to GLP-1 based treatments and in combination with e.g. glucagon or GLP-2 receptor agonistic properties as well (123). Recently, administration of AMG133, has shown promising results in promoting weight loss in initial phase 1 and 2 studies. Paradoxically, these findings indicate that similar results can be obtained by combining GLP-1 receptor activation with either GIP receptor agonism or GIP receptor antagonism. These findings have led to greater interest in understanding the underlying mechanisms of targeting the GIP receptor. Although less than a decade has passed since the first GIP(3-30)NH_2_ injection in humans, substantial research has already contributed to the confirmation of GIP signaling pathways, genetic variations, as well as preclinical and clinical outcomes of GIP receptor targeting compounds (124). Despite the promising therapeutic potential of GIP receptor antagonism, uncertainties remain regarding associated adverse effects. Given that both GIP receptor agonism and antagonism have only been thoroughly investigated in combination with GLP-1 receptor agonism, long-term clinical studies of GIP receptor antagonism alone are needed to evaluate the adverse effect profile.
